# Synthesis and Cytotoxicity Testing of New Amido-Substituted Triazolopyrrolo[2,1-*c*][1,4]benzodiazepine (PBDT) Derivatives

**DOI:** 10.3390/molecules17088762

**Published:** 2012-07-25

**Authors:** Kumaraswamy Sorra, Chi-Fen Chang, Srinivas Pusuluri, Khagga Mukkanti, Min-Chiau Laiu, Bo-Ying Bao, Chia-Hao Su, Ta-Hsien Chuang

**Affiliations:** 1 Medicinal Chemistry Laboratory, GVK Biosciences Private Limited, Plot No. 5C, IDA Uppal, Hyderabad 500039, AP, India; E-Mail: pusulurisrinivas@gvkbio.com (S.P.); 2 Chemistry Division, Institute of Science and Technology, JNT University, Kukatpally, Hyderabad 500072, AP, India; 3 Department of Anatomy and Cell Biology, College of Medicine, National Taiwan University, 1-1, Jen-Ai Road, Taipei 100, Taiwan; E-Mail: changchifen@ntu.edu.tw; 4 Center for Translational Research in Biomedical Sciences, Kaohsiung Chang Gung Memorial Hospital, Kaohsiung 833, Taiwan; E-Mail: a0910758792@hotmail.com; 5 School of Pharmacy, China Medical University, Taichung 40402, Taiwan; E-Mail: bao@mail.cmu.edu.tw

**Keywords:** triazolopyrrolobenzodiazepine, Lawesson’s reagent, cytotoxicity, Mahlavu celles, MRC-5 cells

## Abstract

A series of amido-substituted triazolopyrrolo[2,1-*c*][1,4]benzodiazepine (**PBDT**) derivatives was synthesized from isatoic anhydride, and their cytotoxicity against the MRC-5 and Mahlavu cell lines was evaluated. The results suggest that compound **PBDT-7i** with the *meta*-trifluoromethylbenzoyl substituent can selectively inhibit the growth of Mahlavu cells and has low toxicity towards MRC-5 cells.

## 1. Introduction

Cancer is a leading cause of death globally, accounting for 7.6 million deaths in 2008. Hepatocellular carcinoma (HCC) is one of the most common cancers worldwide and occurs at a high rate (3–5% annually) in patients with chronic viral hepatitis and cirrhosis. HCC is also a major cause of morbidity and mortality in patients with advanced liver disease [1,2]. Therefore, there is a desire to design and develop more potent therapeutic agents for cancer. We are interested in investigating *N*-bridged heterocycles composed of two bioactive heterocyclic moieties, such as benzodiazepines and triazoles, in a single molecular scaffold because such bridged molecules are endowed with a variety of biological activities and have a wide range of therapeutic properties.

**Figure 1:**
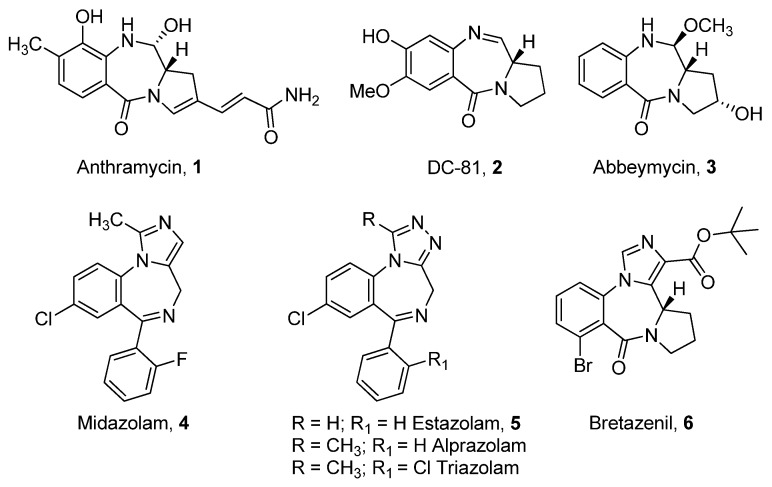
Members of the benzodiazepine family.

Among pharmacologically important heterocyclic compounds, benzodiazepines are useful scaffolds that are widely applied for various therapeutic purposes [3,4,5,6,7,8]. Pyrrolo[2,1-*c*][1,4]benzodiazepines (**PBD**s) are found in natural antitumor antibiotics isolated from *Streptomyces* species; these natural antitumor antibiotics include anthramycin (**1**), tomaymycin, chicamycin A, DC-81 (**2**), mazethramycin, abbeymycin (**3**), neothramycin A and B and porothramycin B (Figure 1) [9]. **PBD**s can recognize and bind to specific DNA sequences and are potential regulators of gene expression. These compounds have possible applications as therapeutic agents for the treatment of genetic disorders including cancer [10]. Benzodiazepines made up of fused tri- and tetraheterocyclic rings have attracted attention in the area of medicinal chemistry; midazolam (**4**), flumazenil and estazolam (**5**) have attracted attention in the area of CNS disorders, and bretazenil (**6**) has emerged for the treatment of neurodegenerative diseases [11,12,13,14,15] (Figure 1).

In addition, 1,2,4-triazole and its derivatives are being used in a variety of therapeutic applications due to their antibacterial, antifungal, anticancer, antitumor, anticonvulsant, anti-inflammatory and analgesic properties [16,17]. 1,2,4-Triazoles fused with other heterocyclic moieties possess a broad spectrum of biological activities [18].

Owing to the biological significance of these two classes of compounds, we planned to synthesize a combined molecular framework. We report herein the synthesis of tetracyclic triazolopyrrolo[2,1-*c*][1,4]benzodiazepines (**PBDTs**) and their amido-substituted derivatives. The cytotoxicity of the newly synthesized derivatives was investigated using the Mahlavu (human hepatocellular carcinoma) and MRC-5 (normal human fibroblast) cell lines.

## 2. Results and Discussion

### 2.1. Synthesis 

**Scheme 1:**
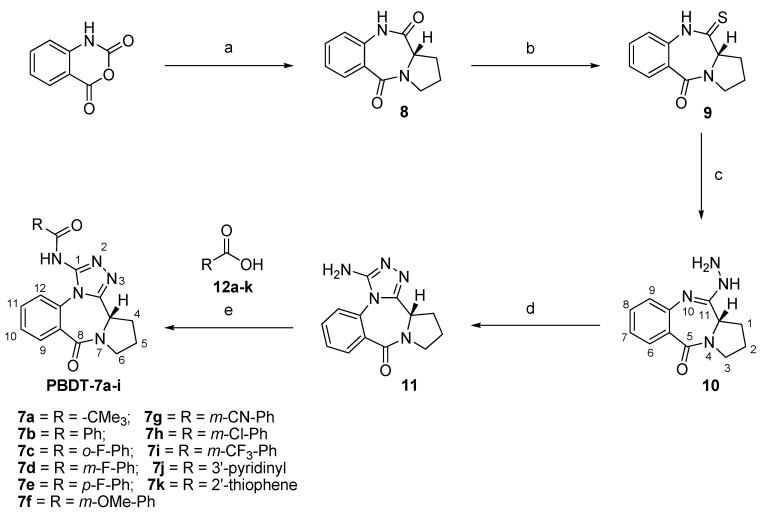
Synthesis of triazolo-fused **PBD** and derivatives.

The synthesis strategy for constructing the tetracyclic 1-amido-substituted triazolopyrrolo[2,1-*c*][1,4]benzodiazepin-8-one derivates **PBDT-7a–k** is shown in Scheme 1. The key intermediate **11** was prepared in four steps. First, pyrrolo-benzodiazepinedione **8** was obtained by the cyclocondensation of L-proline with isatoic anhydride. The corresponding mono-thiolactam **9** was produced by thiation employing Lawesson’s reagent in toluene at 70 °C [19]. Subsequently, the thiolactam **9** was converted into 11-hydrazinopyrrolo[2,1-*c*][1,4]benzodiazepine (**10**) using hydrazine hydrate in ethanol at rt, and cyclization with cyanogen bromide afforded the triazolo **PBD 11** [20,21]. Then, a series of amido-substituted triazolo-fused **PBD**s (compounds **PBDT-7a–k**) was synthesized from the tetracyclic intermediate **11** by coupling these molecules with the corresponding carboxylic acids using HATU as a coupling agent in the presence of Hünig’s base (Table 1).

### 2.2. Cytotoxicity

**Figure 2:**
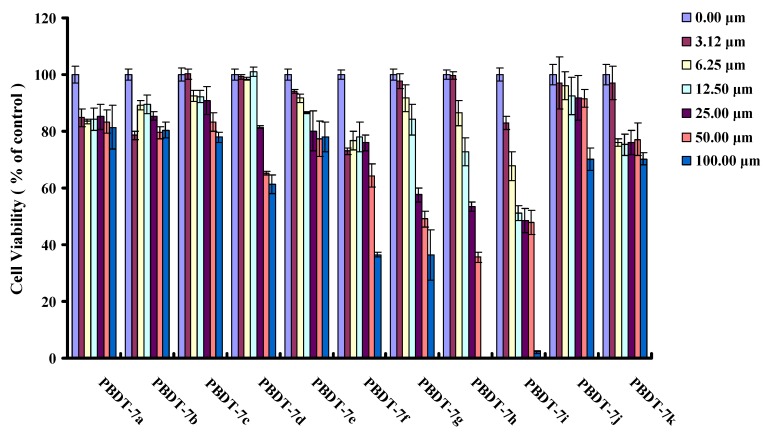
**PBDT-7a–k** induced the death of Mahlavu cells. The concentration-dependent effects of **PBDT-7a–k** on human hepatocellular carcinoma cells. Mahlavu cells were treated with **PBDT-7** series compounds for 48 h, and survival was assessed using a CCK-8 assay (mean ± SEM, n = 6).

**Figure 3:**
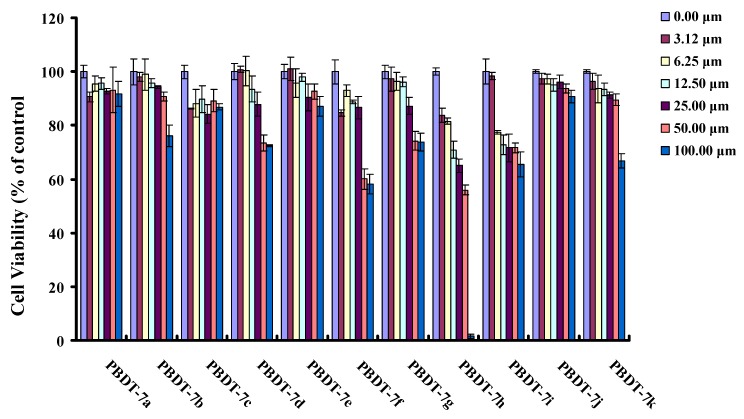
The cytotoxicity assay of **PBDT-7a–k** against MRC-5 cells. The concentration-dependent effect of **PBDT-7a–k** on normal human fibroblast cells. MRC-5 cells were treated with **PBDT-7** series compounds for 48 h, and survival was assessed using a CCK-8 assay (mean ± SEM, n = 6).

The cytotoxic activities of the newly synthesized amido-substituted triazolopyrrolo [2,1-*c*][1,4]benzodiazepines **PBDT-7a–k** were tested against Mahlavu and MRC-5 cells. After incubation of the cells with the compounds at concentrations of 3.12, 6.25, 12.50, 25.0, 50.0 and 100 µM for 48 h, the cell growth and viability were assessed by using CCK-8 assay. The results are shown in Figure 2 and Figure 3. Our initial results showed that percentages of viable Mahlavu and MRC-5 cells exposed to the compounds **PBDT-7a,b** and **PBDT-7j,k** were above 75%, although the concentration was increased to 100 µM. This revealed that the aliphatic and aromatic amides seem no significant difference observed in cytotoxicity between Mahlavu and MRC-5 cell lines. Intriguingly, cells exposed to compounds with various substituents [-F (**PBDT-7c–e**), -OMe (**PBDT-7f**), -CN (**PBDT-7g**), -Cl (**PBDT-7h**) and -CF_3_ (**PBDT-7i**)] on the phenyl ring of the **PBDT** exhibited different viabilities for both cell lines. Compounds with the fluoro substituent at various positions (compounds **PBDT-7c–e**) exhibited different levels of toxicity against Mahlavu and MRC-5 cells. The viability of Mahlavu cells exposed to the compound with a fluoro substituent at the *meta*-position (compound **PBDT-7d**) was approximately 61% at 100 µM, whereas the *ortho*- and *para*-fluoro-substituted compounds **PBDT-7c** and **e** had little effect at the same concentration.

Based on the preliminary results, we selected to further investigate the *meta*-position of the phenyl group (compounds **PBDT-7f–i**). The cell viabilities were reduced in the presence of the chloro-substituted **PBDT-7h** in a dose-dependent manner, and the survival rate of Mahlavu cells was below 50% at 50 µM. **PBDT-7h** was a potent inhibitor of Mahlavu cells, but was also highly toxic for normal cells. To increase the specificity, we evaluated other three *meta*-substituted **PBDT**s, including compounds with the -OMe (**PBDT-7f**), -CN (**PBDT-7g**) and -CF_3_ (**PBDT-7i**) substituents. It should be noted that the cell viabilities of Mahlavu cells were decreased to 37%, 36%, and 2% at 100 µM, respectively, and the IC_50_ values of **PBDT-7g** and **PBDT-7i** were 50 µM for Mahlavu cells. These compounds were slightly toxic to MRC-5 cells, with cell viabilities above 70% at 50 µM. These revealed compound **PBDT-7i** with the *meta*-trifluoromethylbenzoyl substituent can selectively inhibit the growth of Mahlavu cells and is low-toxic to MRC-5 cells. This novel compound certainly deserves further careful investigation to determine its mechanism of action.

## 3. Experimental

### 3.1. General

Melting point (mp) determinations were performed by using a Mel-temp apparatus and are uncorrected. ^1^H- and ^13^C-NMR spectra were recorded in DMSO-*d*_6_ (unless otherwise specified) on a Varian Unity instrument at room temperature at 400/100 MHz, respectively. Chemical shifts are reported in δ parts per million (ppm) downfield from tetramethylsilane (TMS) with reference to internal solvent and coupling constants are expressed in Hz. Mass spectra were obtained on a Micromass QuattroMicro^TM^API-autospectrometer using the APCI technique. HRMS TOF-ES mass spectra were recorded on a Waters-Alliance 2695 Separation Module/Q-TOF Micromass. Infrared (IR) spectra (KBr disks) were recorded on a Perkin Elmer FT-IR spectrometer. Optical rotations ([α]_D_) were measured on a JASCO P-1030 polarimeter. Liquid chromatography-mass spectrometry (LCMS) data was generated on a Waters acquity UPLC photodiode array detector (PDA) system using the ESI technique.

### 3.2. Synthesis

*(11aS)-2,3-Dihydro-1H-pyrrolo[2,1-c][1,4]benzodiazepine-5,11(10H,11aH)-dione* (**8**). A suspension of isatoic anhydride (10.0 g, 61.34 mmol) and L-proline (7.06 g, 61.34 mmol) in *N*,*N*-dimethyl formamide (50 mL) was heated to 140 °C for 5 h. The solvent was removed *in vacuo* and the residue was taken up in water. The precipitate was collected and dried to give **8** (11.30 g, 85% yield) as an off-white solid. mp 214–216 °C; [α]_D_^23^ +497° (*c* 1.03, MeOH); IR *ν_max_* 3200, 1700, 1630 cm^−1^; ^1^H-NMR (CDCl_3_): *δ* 1.97–2.08 (3H, m), 2.72–2.80 (1H, m), 3.56–3.63 (1H, m), 3.77–3.82 (1H, m), 4.07 (1H, d, *J* = 6.2 Hz), 7.04 (1H, d, *J* = 8.0 Hz), 7.23–7.27 (1H, m), 7.45–7.47 (1H, m), 7.99 (1H, dd, *J* = 8.0, 1.6 Hz), 8.92 (1H, br s, NH); ^13^C-NMR (CDCl_3_): *δ* 23.6, 26.4, 47.4, 56.8, 121.2, 125.2, 127.3, 131.3, 132.6, 135.5, 165.6, 171.5; LCMS (ES^+^): *m*/*z* 217 [M+H]^+^; HRMS (TOF ES^+^): MH^+^, calcd for C_12_H_13_N_2_O_2_: 217.0971; found: 217.0972 [M+H]^+^.

*(11aS)-11-Thioxo-1,2,3,10,11,11a-hexahydro-5H pyrrolo [2,1-c][1,4]ben-zodiazepin-5-one* (**9**). To a suspension of dilactam **8** (5.0 g, 23.10 mmol) in dry toluene (300 mL) was added Lawesson’s reagent (4.7 g, 11.55 mmol). The yellow suspension was heated to 70 °C for 5 h. After this time a yellow solid has precipitated which was recrystallized from ethanol to yield pure **9** (4.6 g, 86% yield) as yellow crystals. mp 291–292 °C; [α]_D_^27^ +729.1° (*c* 1.08, DMSO); IR *ν*_max_ 3446, 1616, 1605, 1521, 886 cm^−1^; ^1^H-NMR (CDCl_3_): *δ* 1.97–2.03 (1H, m), 2.08–2.18 (1H, m), 2.22–2.31 (1H, m), 3.08–3.13 (1H, m), 3.57–3.64 (1H, m), 3.77–3.80 (1H, m), 4.22 (1H, d, *J* = 8.0 Hz), 7.05 (1H, d, *J* = 8.0 Hz), 7.33–7.37 (1H, m), 7.49–7.53 (1H, m), 8.04 (1H, d, *J* = 8.0 Hz), 9.73 (1H, br s, NH); ^13^C-NMR: *δ* 22.6, 28.9, 46.8, 59.7, 121.8, 125.6, 127.7, 130.2, 132.1, 136.4, 164.1, 201.9; LCMS (ES^+^) *m*/*z* 233 (M+H^+^); HRMS (TOF ES^+^): MH^+^, calcd for C_12_H_13_N_2_OS: 233.0749; found 233.0738 [M+H]^+^.

*(S)-11-Hydrazinyl-2,3-dihydro-1H-benzo[e]pyrrolo[1,2-a][1,4]diazepin-5(11aH)-one* (**10**). A solution of compound **9** (2.5g, 10.77 mmol) and 98% hydrazine monohydrate (0.65 g, 12.98 mmol) in ethanol (25 mL) was stirred for 4 h at room temperature. The solvent was removed under reduced pressure and the residue was taken up in water. The precipitate was collected, dried and washed with diethyl ether to yield compound **10** (1.74 g, 70% yield) as a white solid. mp 178–180 °C; IR *ν_max_* 3370, 3330, 1640, 1600 cm^−1^; [α]_D_^26^ +154° (*c* 1.02, MeOH). ^1^H-NMR: *δ* 1.78–1.82 (2H, m), 2.14–2.18 (1H, m), 2.46–2.50 (1H, m), 3.02 (1H, d, *J* = 11.2 Hz), 3.34 (1H, d, *J* = 11.2 Hz), 4.62–4.66 (1H, m), 6.32–6.37 (1H, br s, NH), 7.15 (1H, t, *J* = 7.8 Hz); 7.31 (1H, d, *J* = 7.8 Hz); 7.47 (1H, t, *J* = 7.8 Hz); 7.80 (1H, d, *J* = 7.8 Hz); ^13^C-NMR: *δ* 23.6, 26.9, 46.8, 51.0, 122.8, 126.0, 127.0, 129.0, 132.0, 134.2, 151.0, 162.0; LCMS (ES^+^): *m*/*z* 231 [M+H]^+^; HRMS (TOF ES^+^): MH^+^, calcd for C_12_H_15_N_4_O: 231.1205; found 231.1207 [M+H]^+^.

*(13aS)-3-Amino-11,12,13,13a-tetrahydro-9H-benzo[e]pyrrolo[1,2-a][1,2,4]triazolo[3,4-c][1,4]diazepin-9-one* (**11**). A solution of compound **10** (1.0 g, 4.35 mmol) and cyanogen bromide (0.47g, 4.78 mmol) in ethanol (10 mL) was stirred for 12 h at room temperature. The reaction mixture was neutralized with 10% aqueous potassium bicarbonate, the precipitated solid was filtered and recrystallised from EtOH to give pure **11** (0.90 g, 81% yield) as white crystals. mp 210–212 °C; [α]_D_^26^ +104 (*c* 1.02, MeOH); IR *ν_max_* 3339, 1624, 1467, 763 cm^−1^; ^1^H-NMR: *δ* 1.94–1.99 (2H, m), 2.21–2.31 (1H, m), 2.77–2.80 (1H, m), 3.50–3.64 (2H, m), 4.60 (1H, dd, *J* = 8.0, 3.2 Hz), 6.09 (1H, br s, NH), 7.49–7.53 (1H, m), 7.68–7.70 (1H, m), 7.86 (1H, d, *J* = 8.0 Hz); ^13^C-NMR: *δ* 23.2, 26.2, 46.9, 51.1, 122.7, 127.4, 129.6, 130.6, 131.1, 132.1, 150.5, 154.6, 163.7; LCMS (ES^+^): *m*/*z* 256 [M+H]^+^; HRMS (TOF ES^+^): MH^+^, calcd for C_13_H_14_N_5_O: 256.1139; found 256.1135 [M+H]^+^.

*((13aS)-9-Oxo-11,12,13,13a-tetrahydro-9H-benzo[e]pyrrolo[1,2-a][1,2,4]triazolo[3,4-c][1,4]diazepin-3-yl)pivalamide* (**7a**). To a solution of compound **11** (0.1 g, 0.39 mmol) and pyridine (0.2 mL, 2.48 mmol) in T (2 mL) was added pivaloyl chloride (0.058 mL, 47.05 mmol) dropwise at 0 °C. The mixture was stirred at 0 °C for 45 min and filtered. The filtrate was concentrated *in vacuo*, the residue was dissolved in ethyl acetate, washed sequentially with water and saturated sodium bicarbonate solution, dried over sodium sulfate and concentrated under reduced pressure to give compound **7a** (0.086 g, 65% yiled) as an off-white solid. mp 213–215 °C; IR *ν_max_* 3296, 2957, 1687, 1630 cm^−1^; ^1^H-NMR: *δ* 1.11 (9H, s), 2.01–2.04 (2H, m), 2.31–2.39 (1H, m), 2.81–2.89 (1H, m), 3.50–3.65 (2H, m), 4.79–4.83 (1H, m), 7.42 (1H, d, *J* = 8.0 Hz), 7.55 (1H, d, *J* = 8.0 Hz), 7.68 (1H, d, *J* = 6.8 Hz), 7.89 (1H, d, *J* = 6.8 Hz), 10.26 (1H, br s, NH); LCMS (ES^+^): *m*/*z* 340 [M+H]^+^.

### 3.3. General Procedure for Amide Coupling: Preparation of ***7b–k***

To a stirred solution of compound **11** (0.1 g, 0.39 mmol) and *N*,*N*-diisopropylethylamine (0.17 mL, 0.98 mmol) in *N*,*N*-dimethylformamide (2 mL) was added the appropriate carboxylic acid **12b**–**k** (43.14 mmol) and 2-(7-aza-1*H*-benzotriazole-1-yl)-1,1,3,3-tetramethyluronium hexafluorophosphate (HATU) (0.22 g, 0.59 mmol) at room temrature. The reaction mixture was heated at 50 °C for 15–45 min. The mixture was dissolved in ethyl acetate, washed sequentially with water and saturated sodium bicarbonate solution, dried over sodium sulfate and concentrated *in vacuo*. The crude product was purified by column chromatography (SiO_2_, 10-30% ethyl acetate/*n*-hexane).

*N-((13aS)-9-Oxo-11,12,13,13a-tetrahydro-9H-benzo[e]pyrrolo[1,2-a][1,2,4]triazolo[3,4-c][1,4]diazepin-3-yl)benzamide* (**7b**). Yield 80%; off-white solid; mp 173–175 °C; IR *ν_max_* 3442, 1641, 1582, 1332, 699 cm^−1^; ^1^H-NMR: *δ* 2.0–2.05 (2H, m), 2.31–2.40 (1H, m), 2.80–2.85 (1H, m), 3.50–3.74 (2H, m), 4.82–4.84 (1H, m), 7.40–7.62 (6H, m), 7.82–8.00 (3H, m), 11.22 (1H, br s, NH); LCMS (ES^+^): *m*/*z* 360 [M+H]^+^.

*2-Fluoro-N-((13aS)-9-oxo-11,12,13,13a-tetrahydro-9H-benzo[e]pyrrolo[1,2-a][1,2,4]triazolo[3,4-c][1,4]diazepin-3-yl)benzamide* (**7c**). Yield 75%; white solid; mp 180–181 °C; IR ν_max_ 3437, 1647, 1572, 1326, 827 cm^−1^; ^1^H-NMR: δ 1.98–2.03 (2H, m), 2.31–2.40 (1H, m), 2.81–2.89 (1H, m), 3.51–3.72 (2H, m), 4.82–4.84 (1H, m), 7.23–7.38 (1H, m), 7.53–7.72 (5H, m), 7.86–7.94 (2H, m), 11.20 (1H, br s, NH); LCMS (ES^+^): m/z 378 [M+H]^+^.

*3-Fluoro-N-((13aS)-9-oxo-11,12,13,13a-tetrahydro-9H-benzo[e]pyrrolo[1,2-a][1,2,4]triazolo[3,4-c][1,4]diazepin-3-yl)benzamide* (**7d**). Yield 80%; white solid; mp 159–160 °C; IR ν_max_ 3437, 1640, 1579, 1342, 765 cm^−1^; ^1^H-NMR: δ 1.98–2.04 (2H, m), 2.29–2.41 (1H, m), 2.78–2.82 (1H, m), 3.52–3.71 (2H, m), 4.82–4.84 (1H, m), 7.36–7.42 (1H, m), 7.48–7.56 (2H, m), 7.63–7.92 (5H, m); LCMS (ES^+^): m/z 378 [M+H]^+^.

*4-Fluoro-N-((13aS)-9-oxo-11,12,13,13a-tetrahydro-9H-benzo[e]pyrrolo[1,2-a][1,2,4]triazolo[3,4-c][1,4]diazepin-3-yl)benzamide* (**7e**). Yield 75%; white solid; mp 319–321 °C; IR ν_max_ 3435, 1608, 1559, 1338, 778 cm^−1^; ^1^H-NMR: δ 2.00–2.05 (2H, m), 2.31–2.40 (1H, m), 2.82–2.85 (1H, m), 3.50–3.78 (2H, m), 4.82–4.84 (1H, m), 7.20–7.25 (1H, m), 7.38–7.42 (1H, m), 7.55–7.63 (2H, m), 7.92–7.96 (2H, m), 8.12–8.19 (2H, m), 11.23 (1H, br s, NH); LCMS (ES^+^): m/z 378 [M+H]^+^.

*3-Methoxy-N-((13aS)-9-oxo-11,12,13,13a-tetrahydro-9H-benzo[e]pyrrolo[1,2-a][1,2,4]triazolo[3,4-c][1,4]diazepin-3-yl)benzamide* (**7f**). Yield 70%; off-white solid; mp 168–170 °C; IR ν_max_ 3454, 1639, 1590, 1327, 685 cm^−1^; ^1^H-NMR: δ 2.00–2.05 (2H, m), 2.35–2.41 (1H, m), 2.80–2.85 (1H, m), 3.55–3.61 (1H, m), 3.62–3.69 (1H, m), 3.80 (3H, s), 4.82–4.84 (1H, m), 7.15–7.21 (1H, m), 7.39–7.64 (6H, m), 7.89–7.93 (1H, m), 11.21 (1H, br s, NH); LCMS (ES^+^): m/z 390 [M+H]^+^.

*3-Cyano-N-((13aS)-9-oxo-11,12,13,13a-tetrahydro-9H-benzo[e]pyrrolo[1,2-a][1,2,4]triazolo[3,4-c] [1,4]diazepin-3-yl)benzamide* (**7g**). Yield 65%; pale yellow solid; mp 245–246 °C; IR ν_max_ 3435, 2217, 1645, 1535, 651 cm^−1^; ^1^H-NMR: δ 1.98–2.03 (2H, m), 2.31–2.42 (1H, m), 2.76– 2.81 (1H, m), 3.51–3.71 (2H, m), 4.82–4.84 (1H, m), 7.54–7.76 (3H, m), 7.91–8.02 (3H, m), 8.24–8.37 (2H, m); LCMS (ES^+^): m/z 385 [M+H]^+^.

*3-Chloro-N-((13aS)-9-oxo-11,12,13,13a-tetrahydro-9H-benzo[e]pyrrolo[1,2-a][1,2,4]triazolo[3,4-c][1,4]diazepin-3-yl)benzamide* (**7h**). Yield 65%; off-white solid; mp 175–177 °C; IR ν_max_ 3434, 1642, 1565, 1335, 788 cm^−1^; ^1^H-NMR: δ 1.98–2.03 (2H, m), 2.31–2.40 (1H, m), 2.76–2.81 (1H, m), 3.51–3.75 (2H, m), 4.82–4.84 (1H, m), 7.48–7.78 (4H, m), 7.88–7.98 (4H, m); LCMS (ES^+^): m/z 394 [M+H]^+^.

*N-((13aS)-9-Oxo-11,12,13,13a-tetrahydro-9H-benzo[e]pyrrolo[1,2-a][1,2,4]triazolo[3,4-c][1,4]diazepin-3-yl)-3-(trifluoromethyl)benzamide* (**7i**). Yield 70%; white solid; mp 234–236 °C; IR ν_max_ 3431, 1645, 1580, 756 cm^−1^; ^1^H-NMR: δ 2.00–2.05 (2H, m), 2.35–2.41 (1H, m), 2.79–2.82 (1H, m), 3.52–3.60 (1H, m), 3.67–3.75 (1H, m), 4.82–4.84 (1H, m), 7.57–7.60 (1H, m), 7.65–7.75 (2H, m), 7.92–7.98 (3H, m), 8.22–8.35 (2H, m); LCMS (ES^+^): m/z 428 [M+H]^+^.

*N-((13aS)-9-Oxo-11,12,13,13a-tetrahydro-9H-benzo[e]pyrrolo[1,2-a][1,2,4]triazolo[3,4-c][1,4]diazepin-3-yl)nicotinamide* (**7j**). Yield 75%; pale yellow solid; mp 225 °C; IR *ν_max_* 3445, 2923, 2360, 1700, 1628, 1229, 759 cm^−1^; ^1^H-NMR: *δ* 2.00–2.05 (2H, m), 2.37–2.41 (1H, m), 2.78-2.82 (1H, m), 3.52–3.68 (2H, m), 4.82–4.85 (1H, m), 7.42–7.61 (2H, m), 7.91–7.95 (1H, m), 8.21–8.35 (2H, m), 8.67–8.80 (1H, m), 7.92–7.96 (1H, m), 9.12–9.20 (1H, m), 11.55 (1H, br s, NH); LCMS (ES^+^): *m*/*z* 361 [M+H]^+^.

*N-((13aS)-9-Oxo-11,12,13,13a-tetrahydro-9H-benzo[e]pyrrolo[1,2-a][1,2,4]triazolo[3,4-c][1,4]diazepin-3-yl)thiophene-2-carboxamide* (**7k**). Yield 72%; off-white solid; mp 185–186 °C; IR ν_max_ 3425, 1635, 1561 cm^−1^; ^1^H-NMR: δ 2.00–2.05 (2H, m), 2.31–2.40 (1H, m), 2.85–2.92 (1H, m), 3.45–3.65 (2H, m), 4.81–4.83 (1H, m), 7.02–7.22 (1H, m), 7.42–7.62 (3H, m), 7.90–8.20 (3H, m), 11.28 (1H, br s, NH); LCMS (ES^+^): m/z 366 [M+H]^+^.

### 3.4. Biological Activity Test Procedures

#### 3.4.1. Cell Culture

Human fibroblast cells (MRC-5) cells were used for cell culture experiments. Cells were cultured with 90% Eagle’s minimum essential medium with 2 mM L-glutamine and Earle’s BSS adjusted to contain 1.5 g/L sodium bicarbonate, 0.1 mM non-essential amino acids, 1.0 mM sodium pyruvate with 10% fetal bovine serum, 100 U/mL penicillin, and 100 μg/mL streptomycin. The cells were placed in an incubator to maintain high humidity, 37 °C, and 5% CO_2_ for the cell culture experiments. Cells were routinely sub-passaged in a 1:2 to 1:5 split using 0.25% trypsin. Human hepatocellular carcinoma (Mahlavu) epithelial cells were used for cell culture experiments. Cells were cultured with 90% Dulbecco’s modified Eagle’s medium with 10% fetal bovine serum, 100 U/mL penicillin, and 100 μg/mL streptomycin. The cells were placed in an incubator to maintain high humidity, a temperature of 37 °C, and 5% CO_2_ for cell culture experiments. Cells were routinely sub-passaged in a 1:4 to 1:6 split using 0.25% trypsin.

#### 3.4.2. CCK-8 for Cell Proliferation Assay

The cell viability was determined using a CCK-8 assay following the manufacturer’s instructions. Mahlavu cells and MRC-5 cells were seeded in 96-well plates and allowed to attach overnight at 37 °C. Cells were treated with **PBDT-7a–k** in 0.1% FBS MEM or DMEM (100 μL/well), and the cells were incubated for 48 h in a humidified atmosphere. After the incubation period, 10 μL of cell Counting Kit-8 solution was added to the medium, and the cells were incubated for an additional 1–4 h in the incubator. The absorbance at 450 nm was then measured using a microplate reader. The IR was calculated using the following equation: IR = [1 − (A value for the treated samples − A value for the blank samples)/(A value for the control samples − A value for the blank samples)] × 100%. The assays were performed in triplicate and repeated at least twice.

## 4. Conclusions

We have synthesized a series of novel amido-substituted triazolopyrrolo [2,1-*c*][1,4]benzo-diazepines from isatoic anhydride in high total yields. The biological evaluation of these compounds based on the growth of Mahlavu and MRC-5 cells revealed that compound **PBDT-7i** selectively inhibited the proliferation of Mahlavu cells. More interestingly, compound **PBDT-7i** might inhibit not only Mahlavu cell growth, but also shows low toxicity towards MRC-5 cells. 

## Figures and Tables

*Reagents and conditions*: (a) L-Proline, DMF, 140 °C, 5 h, 85% yield; (b) Lawesson’s reagent, toluene, 70 °C, 5 h, 86% yield; (c) 98% N_2_H_4_.H_2_O, EtOH, rt, 4 h, 70% yield; (d) BrCN, EtOH, rt, 12 h, 81% yield; (e) (i) **PBDT-7a**: pivaloyl chloride, pyridine, THF, 0 °C, 45 min; (ii) **PBDT-7b–k**: HATU, *i*-Pr_2_NEt, DMF, 50 °C, 15–45 min.

**Table 1:** Series of triazolo-pyrrolo-benzodiazepine derivatives **PBDT-7a–k** synthesized from compound **11** by coupling with the corresponding carboxylic acids.

S.No.	Acid (12a–k)	PBDT-7	Time (min)	Yield (%)
1	R = *t*-butyl *	**7a**	45	65
2	R = phenyl	**7b**	20	80
3	R = *o*-fluorophenyl	**7c**	30	75
4	R = *m*-fluorophenyl	**7d**	30	80
5	R = *p*-fluorophenyl	**7e**	30	75
6	R = *m*-methoxyphenyl	**7f**	15	70
7	R = *m*-cyanophenyl	**7g**	40	65
8	R = *m*-chlorophenyl	**7h**	30	65
9	R = *m*-trifluoromethylphenyl	**7i**	15	70
10	R = 3′-pyridinyl	**7j**	30	75
11	R = 2′-thiophene	**7k**	30	72

* Pivaloyl chloride was used.
